# Challenges and Limitations of Selective Laser Trabeculoplasty in Modern Glaucoma Management: A Narrative Review

**DOI:** 10.3390/jcm15072691

**Published:** 2026-04-02

**Authors:** Jasmine Okafor, Daniel Laroche

**Affiliations:** Advanced Eyecare of New York, New York, NY 10027, USA

**Keywords:** trabecular, IOP, postoperative, complications, outcomes, progression

## Abstract

Selective laser trabeculoplasty (SLT) has become a mainstay in the management of a variety of glaucoma diseases. SLT provides a safe, minimally invasive, and effective way to lower intraocular pressure. Many studies have discussed its favorable safety profile, and the benefits of SLT being used as a primary and adjunctive therapy in glaucoma. However, while there are many benefits, it is important to note the potential drawbacks that can be seen with this procedure, particularly for non-ideal populations. Common complications include transient episodes of intraocular pressure spikes, hyperemia, blurred vision, and anterior chamber inflammation. Rarer complications such as corneal edema, hyphema, or vision loss have also been reported. This narrative literature review examines the incidence and mechanisms of SLT-related complications, drawing from peer reviewed publications and case reports. The goal is to highlight selective laser trabeculoplasty limitations and complications for physicians to make a more informed decision, counsel patients on possible risks, and implement strategies for optimal outcomes.

## 1. Introduction

Globally, glaucoma is the second leading cause of blindness after cataracts and becomes the leading cause of blindness after the age of 60. Glaucoma is expected to affect 111.8 million people by 2040. The wide reach of this disease poses a significant problem worldwide [1]. Glaucoma is a disease that damages the optic nerve; this is primarily due to the reduced outflow of aqueous humor, leading to elevated eye pressure and the destruction of the nerve [2]. Aqueous humor flows through the trabecular meshwork located in the drainage angle. This process keeps the intraocular pressure (IOP) in the ideal range with a mean normal of 15 mmHg. However, if the trabecular meshwork is not working adequately, this can have complications where the pressure increases, subsequently damaging the nerve [2]. The mean intraocular pressure of untreated glaucoma patients is 18 mmHg [3]. The most common type of glaucoma is Primary Open-Angle Glaucoma (POAG). This occurs when the structure of the angle is visible, but the aqueous fluid does not drain out of the angle properly. Primary Angle Closure Glaucoma (PACG) is the second most common type, and this occurs when an individual’s iris obstructs the trabecular meshwork and aqueous outflow [2].

Selective laser trabeculoplasty (SLT) is a laser treatment used to reduce intraocular pressure in patients with primary open-angle glaucoma and ocular hypertension [4]. It employs a 532 nm, Q-switched, frequency-doubled Nd:YAG laser that delivers short pulses of energy selectively absorbed by melanin granules within pigmented trabecular meshwork (TM) endothelial cells, particularly those in the uveal and corneoscleral meshwork adjacent to Schlemm’s canal [5]. The laser absorption by melanin-containing TM cells occurs without thermal or structural damage to adjacent tissue, initiating biological and chemical responses that remodel the TM and enhance aqueous humor outflow. Morphological studies have shown ablation of TM endothelial cells from the surface of trabecular beams, with the greatest effect in highly pigmented regions, while preserving the underlying beams and the deeper juxtacanalicular tissue, the principal site of outflow resistance [4,5]. This remodeling process likely involves inflammatory or immune-mediated mechanisms, such as monocyte recruitment and cytokine-driven extracellular matrix reorganization [5]. This includes the increased permeability of Schlemm’s canal cells and disintegration of intercellular junctions, all contributing to reduced resistance to aqueous outflow and sustained intraocular pressure reduction [5]. Because SLT acts through cellular and biochemical changes rather than mechanical tissue contraction, it is repeatable and applicable to various forms of open-angle glaucoma. Performed with a nonmagnified, mirrored goniolens, SLT has become an established, minimally invasive, and well-tolerated treatment; however, like all procedures, it carries certain drawbacks despite its generally favorable safety profile [6].

Selective laser trabeculoplasty (SLT) has gained widespread acceptance due to its favorable safety profile, proven efficacy in patients with mild glaucoma, and increasing popularity among ophthalmologists. Over the last decade, SLT has emerged as a preferred treatment for glaucoma because of its effectiveness in lowering intraocular pressure (IOP), the fact that it is a minimally invasive technique, and also because it has demonstrated safety and repeatability in some long-term studies [7]. Large studies have demonstrated a highly favorable safety profile, including the Laser in Glaucoma and Ocular Hypertension trial (LiGHT), which reported no sight-threatening complications among 776 procedures, and a Finnish study of 6081 SLT treatments that identified only a one case of permanent vision loss [8,9]. This technique has been increasingly adopted not only as adjunctive therapy but also as a primary treatment, as it reduces reliance on daily medications and offers a cost-effective option for early disease management. However, despite its advantages and greater efficacy compared to medications in patients with mild glaucoma, SLT’s limitations become more apparent in advanced cases with significant visual field loss. Long-term studies on visual field progression suggest that alternative interventions, such as early cataract extraction or minimally invasive glaucoma surgery (MIGS), may provide additional protection in selected patients against disease progression [4]. For patients over age 50 with ocular hypertension or mild-to-moderate glaucoma who are candidates for cataract surgery, both SLT and combined cataract/MIGS are evidence-based options. However, prior SLT may reduce subsequent MIGS efficacy if cataract surgery is delayed, with studies showing a 53% increased risk of reoperation after angle-based MIGS in eyes with prior laser trabeculoplasty [10]. Cataract/MIGS procedures have increased due to their favorable safety profile and ability to reduce both IOP (1.6–2.3 mmHg beyond cataract extraction alone) and medication burden in a single procedure, while SLT demonstrates superior long-term disease control and cost-effectiveness when used as primary therapy. The choice of treatment should involve shared decision-making that acknowledges these tradeoffs and current evidence limitations, including acknowledgement that most MIGS efficacy data come from industry-sponsored trials. Moreover, SLT carries certain risks, including transient intraocular pressure spikes and other procedure-related complications, underscoring the need for careful patient selection and follow-up. Over the past two decades, the surgical management of glaucoma has evolved, with increasing rates of SLT, cataract surgery, and MIGS. While SLT remains popular as a first-line treatment for open-angle glaucoma due to its moderate success and accessibility, approximately half of SLT-treated patients require additional interventions within 5–10 years, and its IOP-lowering efficacy may be limited in advanced disease or heavily medicated patients [11]. In contrast, early cataract surgery, especially when combined with MIGS, can offer IOP reduction by deepening the anterior chamber, opening the angle, and improving both vision and glaucoma control outcomes that SLT alone may not consistently achieve [12]. Therefore, as more ophthalmologists incorporate SLT into their practice, a thorough understanding of its potential drawbacks, long-term outcomes, and appropriate management strategies remains essential for optimal patient care.

Selective laser trabeculoplasty has also been evaluated in Black and Afro-descendant populations, who experience a higher burden of primary open-angle glaucoma. Studies examining SLT outcomes in these patients suggest that the procedure remains effective and generally well-tolerated, with comparable intraocular pressure reduction and similar rates of transient postoperative complications compared with mixed-ethnicity populations [13]. However, Black populations often present with more advanced glaucoma. Continued investigation into SLT outcomes in diverse populations with more advanced glaucoma is important to better understand treatment response in the long term.

The objective for this literature review is to provide an extensive look into the complications and limitations of selective laser trabeculoplasty. This review aims to provide clarity in the benefits and drawbacks of SLT. This will enable ophthalmologists to decide on the best course of action for creating individualized treatment plans for their glaucoma patients.

## 2. Methods

This study was conducted as a narrative review of the literature that examine the mechanisms, efficacy, and potential complications associated with selective laser trabeculoplasty (SLT) in the treatment of glaucoma.

A literature search was performed using multiple electronic databases, including PubMed, Google Scholar, ScienceDirect, JSTOR, Scopus, and the Cochrane Library. The search strategy utilized combinations of keywords and Medical Subject Headings (MeSH) such as “Selective Laser Trabeculoplasty,” “SLT,” “SLT complications,” “adverse effects,” “glaucoma treatment,” “glaucoma,” “SLT mechanism,” and “laser treatments of glaucoma.” Boolean operators (AND, OR) were used to refine the search strategy.

The search primarily focused on studies published within the past 20 years to ensure relevance to contemporary clinical practice. Eligible publications included peer-reviewed randomized controlled trials, cohort studies, clinical studies, case reports, and systematic reviews reporting on SLT outcomes, mechanisms, safety profiles, or complications. Earlier publications were included when they represented seminal studies that were foundational to the development, mechanisms, or early clinical evaluation of SLT.

Articles were initially screened based on the title and abstract for relevance to the topic. A full-text review was then conducted to determine final eligibility. Reference lists of relevant articles were also manually reviewed to identify additional pertinent studies. Non-peer-reviewed sources were excluded unless they represented established clinical guidelines or consensus statements relevant to glaucoma management.

Relevant data were synthesized qualitatively, focusing on study design, patient population characteristics, SLT treatment parameters, reported complications, and clinical outcomes. Findings were summarized descriptively to highlight clinically relevant pitfalls and considerations associated with SLT therapy.

### 2.1. Overview of SLT Procedure

The mechanisms of SLT are not fully understood. There are multiple proposed theories, but one of the more popular theories is that the laser promotes cellular activity leading to increased recruitment of macrophages in the trabecular meshwork [14]. In addition, cytokine production specifically of IL-1 beta and TNF alpha is further expressed by increasing trabecular stromelysin expression [15]. This, plus the recruitment of macrophages, aids in the remodeling at the trabecular meshwork.

Prior to SLT becoming the mainstay laser for lower intraocular pressure, argon laser trabeculoplasty (ALT) was at the forefront [16]. However, many shortcomings with this method were noted, including high post-treatment IOP spikes, short durations of effective IOP control, post-procedural inflammation, and coagulation at the trabecular meshwork leading to peripheral anterior synechiae [17]. SLT was developed in 1999, and it had a shorter pulse duration and used 1% of the energy of ALT, making it a gentler laser [18].

The indications for SLT are wide and varied, making it very effective in multiple glaucoma diseases. The initial indications for SLT were POAG, pseudoexfoliation, and pigmentary glaucoma. After more research, it was also found to be effective in lowering IOP in ocular hypertension, low-tension glaucoma, steroid-induced glaucoma, and primary angle closure glaucoma with patent iridotomy [18].

It is important to note the potential pitfalls or limitations of SLT in the treatment of glaucoma. First, SLT is not uniformly effective in all patients [19,20]. The magnitude and durability of intraocular pressure reduction are highly variable, with higher baseline IOP being the strongest predictor of response. Some patients, especially those with lower baseline IOP or advanced trabecular meshwork dysfunction, may experience suboptimal or transient benefit, likely due to an insufficient amount of viable TM cells needed to mediate the desired biological response [7,19,20]. Although SLT provides meaningful intraocular pressure reduction, its effect may diminish over time in some patients. SLT is generally considered safe and repeatable. The IOP-lowering effect may be less robust with subsequent procedures, possibly due to cumulative trabecular meshwork cellular exhaustion or altered tissue responsiveness [19,20].

SLT is generally most effective in early glaucoma and may be insufficient as monotherapy in advanced disease requiring lower intraocular pressure targets. A notable proportion of advanced glaucoma patients require subsequent filtering surgery after SLT failure. Patients already on multiple medications often experience limited additional benefit from SLT in lowering IOP or reducing medication burden. While SLT remains safe and can provide some pressure lowering in advanced cases, its ability to bring IOP to sufficiently low levels necessary in more severe disease is often inadequate. This makes more invasive surgical options the recommended treatment for these patients [21]. This decline in efficacy is attributed to the more compromised trabecular meshwork and greater disease progression seen in moderate to advanced glaucoma stages. SLT is generally most successful as an early or adjunctive therapy rather than as a primary treatment in more severe glaucoma or multi-medicated patients [21].

A review by Landers et al. (2021) summarizing six meta-analyses found that selective laser trabeculoplasty reduces intraocular pressure by approximately 20% on average (range 7–36%), with greater reductions observed in patients with higher baseline intraocular pressure [18]. Greater IOP reduction is generally observed in patients with higher baseline pressures [18]. In comparison with topical medications, SLT has demonstrated comparable efficacy in lowering intraocular pressure. A systematic review and meta-analysis by Chi et al. (2020) reported that SLT provides pressure reduction roughly equivalent to slightly more than one glaucoma medication in patients with primary open-angle glaucoma [22].

### 2.2. SLT Complications

Complications associated with selective laser trabeculoplasty range from common transient symptoms reported in large randomized trials to rare vision-threatening events described primarily in isolated case reports. To provide appropriate clinical context, complication rates reported in large clinical trials and cohort studies are summarized in Table 1. Subsequent sections discuss these complications in greater detail, followed by rare events primarily documented in case reports.

### 2.3. Acute Complications

While most adverse events following SLT are mild and transient, as summarized in Table 1, several complications have been reported in the literature. In the LiGHT study of 633 patients who received SLT, 274 reported adverse events. Ten patients experienced a transient IOP spike of more than five, three had inflammation after the procedure, and notably, 241 of 633 patients (38.1%) reported temporary symptoms. including blurred vision, discomfort, photophobia, and hyperemia. Although these symptoms are self-limiting and typically resolve within several days, this relatively high incidence is clinically relevant given that SLT is performed as an outpatient procedure. Patients should therefore be informed that transient visual disturbance, photophobia, discomfort, and redness are common following treatment and are thought to result from transient anterior chamber inflammation [23]. It is important to note that the incidence of elevated IOP after SLT is low [17]. Post-SLT inflammation typically appears within 2–3 days of the procedure and is transient, generally resolving within 5 days. A risk factor for both IOP spikes and inflammation is a heavily pigmented trabecular meshwork [16]. Conjunctival hyperemia is a self-limiting adverse outcome that involves the blood vessels of the eye becoming dilated and engorged [24].

### 2.4. Subacute and Delayed Complications

While most IOP spikes are short term and respond to medical intervention, persistent elevation of IOP or failure to control the IOP can occur. Studies reporting persistent increased IOP primarily involved patients with heavily pigmented eyes or a history of laser treatment [25]. In the case of sustained elevated IOP, surgical intervention may be needed [25].

There have been reports of retinal complications from SLT. These include cystoid macular edema (CME) in patients with existing pseudophakic macular edema [17]. Wechsler et al. (2010) reported a case of CME after a complicated cataract case, and Ha et al. (2014) noted CME with a patient who had moderate nonproliferative diabetes [26,27].

Hyperemia and hyphema are both conditions involving blood in the eye. In a case report from Shihadeh et al. (2006) [28] of a 77-year-old male with POAG, hyphema had occurred post-SLT in the left eye. The episode resolved spontaneously with no sequelae, and IOP control was achieved [28].

Chen et al. (2025) [29] conducted a systematic review and meta-analysis on the long-term efficacy and safety of selective laser trabeculoplasty (SLT). The paper highlighted several pitfalls associated with SLT in the management of primary open-angle glaucoma. Key pitfalls include the risk of transient intraocular pressure (IOP) elevation shortly after the procedure, which may require additional management [29]. Complications such as anterior chamber inflammation, pain, redness, and rare but serious adverse events like cystoid macular edema, hyphema, corneal edema, choroidal effusion, foveal burns, and refractive shifts (both hyperopic and myopic) were reported [29]. The article also notes variable efficacy in patients with different types of glaucoma and high myopia, emphasizing caution in such cases. There is recognition that despite SLT’s general safety and effectiveness, it is not devoid of potentially vision-threatening complications [29]. The authors recommend careful patient selection, lower energy settings for predisposed patients, and unilateral treatment to mitigate risks [29]. Overall, the pitfalls center on post-procedural inflammation, pressure spikes, rare but serious ocular complications, and the need for continued vigilance and patient counseling about risks.

### 2.5. Rare or Severe Complications

Regina et al. (2011) [30] reported two cases of corneal stromal haze developing within 24 to 48 h after SLT, treated with topical steroids for several weeks. The corneal edema resolved, but both patients were left with residual corneal scarring and thinning, and one patient experienced a significant hyperopic shift [30]. Knickelbein et al. (2014) [31] documented four women from three clinical sites who developed acute corneal edema and haze within 2 days of uneventful SLT. In the following weeks to months, all treated corneas thinned below pre-procedure thicknesses, with resultant hyperopic shifts ranging from nearly 2.0 diopters to greater than 6.0 diopters. All eyes were moderately to highly myopic prior to SLT (spherical equivalent from −5.00 to −12.5 D) [31]. Ojanen et al. (2025) [9] reported one myopic eye with permanent vision loss due to corneal scarring, thinning and irregular astigmatism with a hyperopic shift (5.3 D with recovery to 1.25 D in three years), resulting in poor visual acuity (from preoperative 0.9 to 0.4). The incidence was less than 0.2/1000 for all SLT treatments and 1/648 for SLT in high myopia (spherical equivalent ≤ −5.00 D) [9]. Additionally, a case report described bilateral subretinal fluid developing within 24 h of SLT that resulted in permanent retinal pigment epithelial changes and subjectively and objectively decreased best-corrected visual acuity, despite resolution of the subretinal fluid within 4 days [32].

One of the first cases of severe iritis and choroidal effusion was discussed in a case report in 2008 from Kim and Singh. A 72-year-old patient had undergone SLT in both eyes and developed a severe anterior chamber reaction, shallow anterior chamber, and choroidal effusion [33]. In another case reported by Pardines et al. (2016) a 73-year-old woman with uncontrolled open angle glaucoma underwent SLT in both eyes, and developed choroidal effusions in both eyes [34].

Severe bleeding after SLT for glaucoma is extremely rare. The actual incidence of bleeding complications is low. The Ojanen et al. (2025) [9] study of 6081 SLT procedures found that 12 patients had bleeding in the anterior chamber angle (incidence 2/1000 treatments), and only 4 developed hyphema (incidence 0.7/1000 treatments). Importantly, there were no cases of permanent vision loss due to bleeding in this large cohort. The single case of permanent vision deterioration was due to corneal scarring in a highly myopic patient, not bleeding [9].

Vision loss due to IOP spikes following selective laser trabeculoplasty is exceedingly rare, to our knowledge there is no documented cases of permanent vision loss from post-SLT IOP elevation in major clinical trials [23]. Post-procedure IOP spikes typically occur within hours and are transient. A Cochrane systematic review confirmed that perioperative medications significantly reduce the risk of IOP increases of 10 mmHg or greater within two hours (RR 0.05, 95% CI 0.01 to 0.20) [34]. The guideline recommends performing IOP checks within 30 min to 2 h after surgery, and states that “medications that are not being used chronically may be used perioperatively to avert temporary IOP elevations, particularly in those patients with severe disease” [35].

### 2.6. Visual Field Progression

Visual field (VF) progression rates are a crucial metric in evaluating glaucoma treatments, including selective laser trabeculoplasty. The LiGHT trial’s 6-year analysis evaluated visual field progression using hierarchical linear mixed-effects modeling to account for perimetric learning and test–retest variability. The mean deviation progression rate was −0.26 dB/year in the SLT-first arm compared with −0.37 dB/year in the drops-first arm. This represents a 29% slower rate of visual field decline in the SLT first-group [23]. In contrast, the earlier 3-year analysis focused on pointwise total deviation (TD) progression rather than MD, reporting a higher proportion of eyes with moderate or fast TD progression in the medical therapy group (26.2%) vs. the SLT group (16.9%), without specifying the annual decline rates previously cited [36]. Across LiGHT trial analyses, SLT-first treatment was associated with slower visual field progression compared with drops-first therapy. The 6-year data suggest improved visual field preservation compared with initial medical therapy (fewer eyes showing progression: 19.6% vs. 26.8%) [23]. Notably, only 14% of SLT-treated eyes experienced rapid progression (>−0.5 dB/year) vs. 25% in the medication group, with the statistical significance most marked in patients with ocular hypertension or early open-angle glaucoma [23]. These findings support SLT’s role as an effective first-line therapy to preserve visual function over time in appropriately selected patients.

Swain et al.’s (2023) retrospective study of 51 eyes where SLT was used as adjunctive therapy in patients already on medical treatment found the worsening of mean deviation from −5.61 ± 3.90 dB at baseline to −7.64 ± 6.57 dB at 5 years in eyes that did not require subsequent surgical intervention [37]. Pattern standard deviation also significantly increased from 4.63 ± 2.70 dB pre-SLT to 6.84 ± 2.62 dB at 5 years (*p* ≤ 0.01) [37]. Visual field loss continued despite SLT treatment, though it is important to note this was a small, single-center retrospective analysis where SLT was used as adjunctive therapy in patients already on medical treatment [37]. When compared with other surgical interventions, such as combined cataract extraction plus minimally invasive glaucoma surgery (MIGS) like the Hydrus Microstent, the five-year VF progression rate is lower, around −0.26 dB/year per the HORIZON trial [38]. Observational data from surgical trials suggest that cataract extraction combined with minimally invasive glaucoma surgery (MIGS) may provide sustained intraocular pressure reduction and visual field preservation in selected patients. In a prospective study by Fea et al. (2017) and colleagues comparing standalone Hydrus microstent implantation in SLT for 56 patients with uncontrolled primary open-angle glaucoma, both treatments significantly reduced IOP without serious adverse events at 12 months, but the Hydrus demonstrated superior medication reduction [39]. The Hydrus group achieved a 3-fold greater reduction in medication use compared to SLT (−1.4 ± 0.97 vs. −0.5 ± 1.05 medications, *p* = 0.001), with 47% of Hydrus patients medication-free at 12 months compared to only 4% in the SLT group. Both groups experienced significant IOP reduction from baseline, though the study noted that the Hydrus group had more advanced disease at baseline (mean deviation −8.43 ± 6.84 dB vs. −3.04 ± 0.65 dB, *p* < 0.001) [39]. The safety profile favored SLT, with no complications recorded in the SLT group, while the Hydrus group experienced three cases of temporary visual acuity reduction and two postoperative IOP spikes that resolved within one week [39].

Direct randomized head-to-head comparisons between these surgical approaches and SLT remain limited, and the optimal intervention may vary depending on disease severity, treatment goals, and patient characteristics. These findings suggest that while both modalities effectively lower IOP, the Hydrus microstent may offer greater medication independence, though at the cost of a minimally invasive surgical procedure with associated risks. Therefore, SLT, MIGS, and lens-based surgical interventions should be viewed as complementary treatment options within the broader glaucoma management algorithm rather than directly interchangeable therapies. Overall, these data emphasize the benefits of SLT in reducing intraocular pressure and slowing functional decline, but also highlight its limitations particularly in patients with more advanced disease where slower progression and better preservation of visual field may be achieved with combined surgical options. Such nuances emphasize the importance of personalized treatment planning based on disease severity and patient-specific risk factors.

### 2.7. Contraindications, Limitations, and Patient Selection Considerations for Selective Laser Trabeculoplasty

#### Absolute Contraindications

Selective laser trabeculoplasty is traditionally contraindicated in neovascular glaucoma due to the presence of abnormal blood vessels in the anterior chamber angle. Active intraocular inflammation is also considered a contraindication, as laser treatment may exacerbate inflammatory activity. Additionally, dense media opacities that prevent adequate visualization of the trabecular meshwork may preclude safe and effective SLT treatment. Adequate visualization of the trabecular meshwork is essential for accurate laser application and treatment efficacy [40].

### 2.8. Relative Contraindications and Patient Populations with Reduced Response

Although SLT has historically been approached with caution in uveitic glaucoma, it is not considered an absolute contraindication when inflammation is well-controlled. Recent evidence demonstrates that SLT can be safe and effective in uveitic glaucoma, with one 2025 study showing significant intraocular pressure reduction from 28.4 ± 6.50 mmHg to 17.3 ± 9.4 mmHg at six months without significant increase in inflammation or prednisolone dosage. Treatment success was greater in patients with shorter uveitis duration and lower baseline intraocular pressure [41].

Several patient populations may demonstrate reduced response to SLT or require careful consideration prior to treatment. Selective laser trabeculoplasty may be less suitable for patients with moderate to advanced glaucoma, as these patients often require lower intraocular pressure targets than SLT alone can reliably achieve. The Advanced Glaucoma Intervention Study demonstrated that preserving vision in advanced glaucoma requires aggressive intraocular pressure control, with traditional incisional surgeries such as trabeculectomy and glaucoma drainage devices remaining among the most effective interventions [42]. Although SLT is not contraindicated in advanced disease, it may be insufficient as monotherapy and patients should be counseled regarding the potential need for additional surgical interventions.

Eyes with heavily pigmented trabecular meshwork may also experience an increased risk of transient postoperative intraocular pressure spikes due to greater absorption of laser energy. Postoperative IOP spikes have been reported in 4.5% to 27% of eyes, depending on how spike thresholds are defined [43].

Emerging evidence suggests that sex-based differences may influence response to selective laser trabeculoplasty. Analysis of the Laser in Glaucoma and Ocular Hypertension (LiGHT) Trial demonstrated that female sex was negatively associated with early absolute intraocular pressure reduction after SLT (coefficient −0.63; 95% CI, −1.23 to −0.02; *p* = 0.04) [44]. Similarly, data from the Lausanne Laser Trabeculoplasty Registry identified male sex as a positive predictor of treatment success (OR = 2.79; *p* = 0.02) [45]. A 2024 post hoc analysis of the LiGHT China Trial also found that patients nonresponsive to both initial and repeat SLT were more likely to be female, older, and have lower baseline intraocular pressure (difference 27.5%; *p* = 0.03) [43]. These findings suggest that female patients may demonstrate lower treatment responsiveness in some settings and may benefit from closer monitoring after SLT. However, the mechanisms underlying these sex differences remain unclear and warrant further investigation.

### 2.9. General Limitations of Selective Laser Trabeculoplasty

Despite its favorable safety profile, selective laser trabeculoplasty has several inherent limitations. The intraocular pressure-lowering effect of SLT is not uniform across all patients, and treatment response may vary depending on disease severity, baseline intraocular pressure, and trabecular meshwork function. As noted by Leahy et al. (2015), “the treatment is not uniformly effective in all eyes, and its IOP-lowering effect decreases over time” [20].

In addition, mild transient postoperative inflammation may occur. In the West Indies Glaucoma Study, anterior chamber cells were observed in 40.3% of eyes following SLT, with mean cell scores rising significantly during the first postoperative week before returning to baseline levels [46].

These transient complications are largely attributable to the biological response of the trabecular meshwork to laser energy. Selective laser trabeculoplasty induces a controlled inflammatory cascade involving cytokine release and macrophage recruitment, which promotes trabecular meshwork remodeling and improved aqueous outflow [17]. However, this same inflammatory response may also contribute to temporary anterior chamber inflammation and short-term visual symptoms following treatment. Transient intraocular pressure spikes are thought to result from temporary trabecular meshwork edema and accumulation of cellular debris that briefly impede aqueous outflow. Certain patient characteristics, including heavily pigmented trabecular meshwork, high myopia, and advanced glaucoma with a limited intraocular pressure reserve, may increase the likelihood of these complications.

Another limitation involves uncertainty regarding optimal treatment parameters. Currently, no high-level evidence definitively establishes ideal laser energy settings or the optimal timing for repeat SLT treatments, highlighting the need for individualized treatment planning and continued research in this area [11].

Table 2 summarizes the limitations of selective laser trabeculoplasty. Proper patient selection and counseling remain essential to managing expectations regarding potential treatment response and the possible need for additional therapies or interventions. Selective laser trabeculoplasty may be less suitable for patients with moderate to advanced glaucoma, as these patients often require lower intraocular pressure targets than SLT alone can reliably achieve.

### 2.10. Long-Term Outcomes and Monitoring After SLT

Selective laser trabeculoplasty provides meaningful intraocular pressure reduction for many patients, but the durability of its effect may diminish over time. Meta-analytic data indicate that approximately 45% to 98% of treated eyes maintain therapeutic intraocular pressure reduction at one year, declining to approximately 28% to 85% by three years [18]. Long-term follow-up studies also demonstrate a gradual need for additional therapy in some patients. For example, Ansari et al. (2021) reported that 89% of eyes required no additional treatment at two years, decreasing to 60% at five years and 42% at ten years, illustrating the progressive reduction in treatment durability in some patients [47].

Selective laser trabeculoplasty should not be considered a curative treatment for glaucoma, as its intraocular pressure lowering effect may diminish over time and additional therapy may become necessary. Some patients may mistakenly assume that SLT permanently resolves their disease and subsequently discontinue follow-up care. However, ongoing monitoring is essential to detect treatment failure, rising intraocular pressure, or disease progression. Without regular ophthalmic surveillance, patients remain at risk for continued optic nerve damage and progressive visual field loss. Loss to follow-up has been shown to independently increase the risk of vision impairment and blindness in glaucoma, particularly among vulnerable populations where gaps in care are more common [48].

In the LiGHT trial, approximately 69.8% of SLT-treated eyes maintained target intraocular pressure without escalation at six years, while 30.2% required additional medical or surgical treatment, including trabeculectomy in a small number of patients [23]. Although SLT can reduce medication burden and improve adherence when successful, clinical guidelines emphasize that glaucoma remains a chronic disease requiring continued monitoring. The American Academy of Ophthalmology recommends follow-up within six weeks after treatment, with earlier evaluation in patients with advanced disease or optic nerve concerns [49]. These findings highlight the importance of patient education and adherence to long-term follow-up after SLT to preserve visual function.

### 2.11. Prevention and Management of Complications

Management strategies for selective laser trabeculoplasty complications are well established. Perioperative glaucoma medications effectively reduce the risk of intraocular pressure spikes [5]. Postoperative IOP elevations are typically managed with prophylactic glaucoma drops, with alpha-2 agonists such as apraclonidine and brimonidine commonly used [5]. The review by Zhang et al. (2017) demonstrated that perioperative medications significantly reduce the risk of IOP increases of 10 mmHg or greater within two hours, with continued benefit between two and 24 h for both 5 mmHg and 10 mmHg elevations [35]. Alpha-2 agonists (apraclonidine and brimonidine) are commonly used, with brimonidine shown to be as effective as apraclonidine in preventing immediate IOP elevation [11]. IOP checks should be performed from 30 min to 2 h after surgery.

Evidence regarding routine use of topical corticosteroids or NSAIDs after SLT is mixed. The American Academy of Ophthalmology reports level I evidence that these agents do not interfere with IOP-lowering effects. However, the Steroids After Laser Trabeculoplasty (SALT) trial demonstrated greater IOP reduction at 12 weeks in both NSAID and steroid groups compared with placebo [50]. Another study reported that patients in the prednisolone acetate 1% group had higher 12-month success rates (83.7%) compared to other anti-inflammatory regimens (prednisolone acetate 0.12% = 63.9%; ketorolac tromethamine 0.5% = 67.0%; *p* = 0.003) [51].

Earlier RCTs (randomized controlled trials), including De Keyser et al. (2017) and Jinapriya et al. (2014), found no significant benefits for pain, redness, cells, or IOP changes [52,53]. Mild, transient inflammation occurs in ~40% of eyes, with symptoms like hyperemia and discomfort typically self-resolving within a week without intervention [52,53]. While prophylactic IOP-lowering drops effectively reduce spikes, anti-inflammatory prophylaxis lacks strong support overall, emphasizing conservative management and patient education for early intervention.

### 2.12. Alternative Treatment Strategies

When selective laser trabeculoplasty is contraindicated, ineffective, or insufficient to achieve target intraocular pressure, several alternative treatment strategies may be considered. Topical intraocular pressure lowering medications remain widely used first-line therapies, and long-term adherence to glaucoma medications can be challenging for many patients. Minimally invasive glaucoma surgery (MIGS), particularly when combined with cataract surgery, may provide additional pressure reduction in patients with mild-to-moderate glaucoma. In cases of advanced disease or when less invasive treatments fail, traditional filtering procedures such as trabeculectomy or glaucoma drainage device implantation remain the most effective options for achieving substantial intraocular pressure reduction. The choice among these treatment approaches should be individualized based on disease severity, patient adherence, and surgical candidacy.

## 3. Conclusions

In conclusion, selective laser trabeculoplasty (SLT) is a safe and effective option for lowering intraocular pressure in many patients with glaucoma. Most complications associated with SLT are transient and mild, including hyphema, corneal edema, postoperative inflammation, and intraocular pressure spikes, which are generally manageable with appropriate monitoring. However, risk may be slightly higher in certain patient populations, highlighting the importance of individualized counseling and careful patient selection when considering SLT.

SLT appears to be most effective in early glaucoma, where meaningful intraocular pressure reduction can often be achieved with reduced medication burden. However, this can wane over time and patients still require surveillance and many will require medical therapy. In contrast, patients with moderate to advanced disease or those already receiving multiple medications may require additional interventions, including filtering surgery, to achieve target intraocular pressure.

Further research is needed to better identify predictors of treatment response and complication risk, as well as to clarify the role of SLT relative to other treatment strategies such as lens extraction or minimally invasive glaucoma surgery in long-term glaucoma management.

## Figures and Tables

**Table 1:** Reported incidence of complications after selective laser trabeculoplasty.

Complications	Reported Incidence	Source
Transient Discomfort; Photophobia; Hyperemia	34.4%	Laser in Glaucoma and Ocular Hypertension (LiGHT) trial
IOP spike > 5 mmHg	0.8% (6/776 procedures)	LiGHT trial
Hyphema	0.7 per 1000 treatments	Ojanen et al. (2025) [9]
Corneal Edema	~1 per 1000 treatments	Ojanen et al. (2025) [9]
Permanent Vision Loss	<0.2 per 1000 treatments	Ojanen et al. (2025) [9]

**Table 2:** Pitfalls of SLT.

Complication	Event Frequency	Risk Factors	Level of Evidence	Clinical Significance	Key Sources
Transient IOP Spikes	~0.8% (>5 mmHg spike in LiGHT trial)	Heavily pigmented TM, high baseline IOP	Level I (RCT)	Mild– Moderate (usually transient)	LiGHT Trial
Transient inflammation/ hyperemia	~34% transient symptoms	Laser energy, pigment load	Level I–II	Mild	LiGHT Trial
Hyphema	~0.7 per 1000 treatments	Fragile angle vessels	Level III	Mild– Moderate	Ojanen et al. (2025) [9]
Corneal Edema	~1 per 1000 treatments	High myopia, corneal susceptibility	Level III	Moderate	Regina et al. (2011) [30]; Knickelbein et al. (2014) [31]
Corneal Scarring/ hyperopic shift	Rare (<0.2/1000)	High myopia	Level III	Severe	Ojanen et al. (2025) [9]
CME	Rare	Diabetes, prior macular edema	Level III	Moderate	Wechsler et al. (2010) [26]; Ha et al. (2014) [27]
Choroidal effusion	Rare case reports	Inflammatory response	Level III	Moderate– Severe	Kim et al. (2008) [33]; Pardines et al. (2016) [34]
Variable Treatment Durability	Long-term decline in effect	Disease severity, TM damage	Level I–II	Moderate	LiGHT trial; Ansari et al. (2021) [47]

## Data Availability

No new data was created or analyzed in this study. Data sharing is not applicable to this article.
